# Schizophrenia endophenotypes: a review of neurophysiological, neuropsychological, and social cognition markers

**DOI:** 10.3389/fpsyt.2026.1740394

**Published:** 2026-03-10

**Authors:** Ricardo R. Garcia, Francisco Aliste, Guillermo Soto

**Affiliations:** 1 Department of Linguistics and Center for Cognitive Studies, Universidad de Chile, Santiago, Chile; 2 Community Psychiatry Unit, Hospital Padre Hurtado, Santiago, Chile

**Keywords:** attention, ERPs, event-related potentials, executive function, mismatch negativity, MMN, oculomotor impairments, P300

## Abstract

**Introduction:**

In schizophrenia (SCZ) research, endophenotypes represent intermediate links between the polygenic architecture of the disorder and clinical phenomenology. These neurobiological markers must meet specific criteria, including heritability, state independence, and cosegregation within families. This review examines the evolution of endophenotype research, from physiological and cognitive markers to social cognition.

**Method:**

We conducted a narrative mini-review to evaluate recent evidence on the validity of neurophysiological, neuropsychological, and social-cognitive parameters as candidate endophenotypes for SCZ.

**Results:**

Ample evidence supports oculomotor, event-related potentials, and cognitive deficits as endophenotypes found consistently in probands and first-degree relatives. In social cognition, results are more heterogeneous. While Theory of Mind and emotion processing show promise as candidate endophenotypes, evidence regarding social perception and attributional bias remains inconsistent.

**Discussion:**

Current data confirm the utility of neurophysiological and neuropsychological markers as established endophenotypes. While specific social cognition components require further validation, recent investigations demonstrate greater impairments in SCZ on mentalization tasks compared to other psychiatric conditions. The integration of these markers is relevant for stratified psychiatry and treatment personalization. Furthermore, recent findings suggest a transdiagnostic role for certain endophenotypes, indicating shared neural vulnerabilities across the schizoaffective spectrum.

## 1 Introduction

Endophenotypes are a critical component of schizophrenia (SCZ) research, reflecting the disease’s complex inheritance patterns (1). Unlike Mendelian phenotypes, which typically reflect specific genetic liabilities, polygenic disorders such as SCZ involve multiple genes and molecular pathways. Consequently, the biological basis of endophenotypes has become a major subject of investigation in SCZ and bipolar disorder, particularly in light of the reliance on clinical observation for psychiatric diagnostics. While initially focused on biological markers, endophenotype research has expanded to include neuropsychological parameters and, more recently, the emerging domain of social cognition (SC). In this context, endophenotype research has helped elucidate the dynamic interplay among genetic, environmental, and epigenetic factors that collectively drive the pathogenesis of SCZ (1–3). To address this complexity and validate the relevance of phenotypes in studying the genetic basis of SCZ, specific criteria have been proposed for endophenotype identification: 1) association with the illness in the population; 2) heritability; 3) state-independence (manifesting whether the illness is active or in remission); 4) co-segregation with the illness within families; and 5) higher prevalence in unaffected family members compared to the general population (1, 4, 5). This review synthesizes recent evidence on SCZ endophenotypes, focusing primarily on established neurophysiological and neuropsychological findings typically reported in clinical settings. Conversely, neuroimaging and functional connectivity research are not addressed, as these modalities are not currently applied in routine clinical practice for the diagnosis, treatment, or rehabilitation of individuals with SCZ. Furthermore, we aim to assess recent research suggesting that SC may serve as a novel endophenotype, reflecting the increasing recognition of social cognitive deficits in SCZ research. This review begins by examining neurophysiological and neuropsychological markers, followed by an evaluation of recent research proposing SC as a novel candidate endophenotype.

## 2 Neurophysiological endophenotypes in SCZ

In recent years, neurophysiological endophenotypes have become a focus of attention in SCZ research, reflecting developmental alterations in cortical circuits (6). This section discusses physiological biomarkers identified as candidate endophenotypes, including oculomotor impairments and event-related potentials (ERPs) such as Mismatch Negativity (MMN) and P300. These measures were selected for their distinct relevance to mental health practice, where they are increasingly used in routine patient evaluations to support the diagnostic confirmation of SCZ.

### 2.1 Oculomotor impairments

Oculomotor deficits have historically played a pivotal role in SCZ research, both in clinical practice and, later, in laboratory tests. Notably, eye-tracking abnormalities have been consistently linked to SCZ (1, 7, 8). Smooth pursuit eye movements have been proposed as a biomarker for detecting genetic vulnerability to schizophrenia spectrum disorders (9, 10). Subsequent studies have corroborated that oculomotor deficits, particularly in smooth pursuit, constitute valid endophenotypes of SCZ (11). Investigations involving individuals with SCZ and their family members have further substantiated these neurophysiological alterations. The use of novel paradigms, such as foveal stabilization experiments—locking the target to the retina—has demonstrated that individuals with SCZ and their unaffected relatives share deficits in predictive pursuit (9). This experimental paradigm, designed to isolate the predictive processes driving the extraretinal signal, revealed smooth pursuit deficits in disorganized schizotypy, suggesting that predictive pursuit may represent a specific oculomotor endophenotype within the SCZ spectrum (12). Moreover, recent evidence derived from individuals with SCZ and those at ultra-high risk (UHR) for psychosis indicates that SCZ subjects perform significantly worse than controls on the antisaccade (AS) task—which requires looking opposite to a visual cue. Conversely, the memory-guided saccade (MGS) task—executing a saccade to a remembered location—yielded higher error rates across subjects with SCZ, UHR individuals, and siblings. The authors attributed these findings to the specific sensitivity of the MGS task (13), reflecting an oculomotor pattern characterized by impaired inhibitory control. This deficit is likely mediated by neural dysfunction within the dorsolateral prefrontal cortex and the frontal eye fields (14, 15).

### 2.2 Event-related potentials

Neurophysiological investigations provide a framework for examining specific alterations in neural responses to sensory stimuli. Comparative studies utilizing auditory oddball paradigms—sequences of frequent standard tones interspersed with rare deviants—across individuals with SCZ, healthy controls, and first-degree relatives suggest that ERPs may serve as biomarkers of familial risk. However, deficits in the N1 and P3b components appear to be more specific to probands (16). Additional ERP research has focused on MMN and P300 deficits (6). MMN is defined as a negative potential shift occurring 50–200 ms following the presentation of a deviant stimulus within a sequence of repetitive standards. MMN deficits are well-documented in individuals with SCZ (17, 18). Furthermore, attenuated MMN responses have been observed in unaffected relatives (19) and in UHR individuals (20); in the latter group, MMN responses appear to hold predictive value for the transition to full-blown psychoses and SCZ. Finally, the P300—a positive potential elicited approximately 300 ms after a deviant stimulus—also exhibits abnormalities in SCZ, characterized specifically by reduced amplitude and increased latency (21).

### 2.3 Prepulse inhibition deficits

PPI is defined as the physiological attenuation of the startle response to an intense acoustic stimulus when it is immediately preceded by a weaker stimulus, or ‘prepulse’ (6). This sensorimotor gating phenomenon, recorded by electromyography of the orbicularis oculi muscle, is significantly less intense in individuals with SCZ compared to healthy controls (22, 23). Notably, PPI deficits have also been documented in unaffected first-degree relatives (FDRs) of SCZ probands, suggesting a genetic underpinning to these abnormalities (24). However, PPI impairment is not specific to the SCZ spectrum; comparable deficits have been identified in other neuropsychiatric conditions, including obsessive-compulsive disorder, attention deficit hyperactivity disorder, and Huntington’s disease (24, 25).

## 3 Neuropsychological endophenotypes in SCZ

SCZ is marked by progressive cognitive decline, encompassing impairments in verbal memory, attention, working memory (WM), and executive function (26, 27). Large-scale investigations have corroborated significant deficits across these neuropsychological endophenotypes (28). Key aspects of these domains are examined below.

### 3.1 Attentional deficits

Attentional impairments are a well-documented core feature of SCZ, particularly characterized by deficits in sustained attention (6). The Continuous Performance Test (CPT)—which identifies specific targets within a rapid stream of stimuli—has established itself as a robust tool for the reliable assessment of these functions (6). Individuals with SCZ exhibit altered CPT performance; consistent with endophenotype criteria, these deficits are also observed in FDRs, although conflicting evidence has been reported (29). Furthermore, specific CPT variants, such as the Degraded CPT (DPT), which manipulates perceptual load, and the Identical Pairs CPT (CPT-IP) (30, 31), which engages WM, have consistently revealed deficits in SCZ. Notably, a meta-analysis of these studies yielded a mean effect size of 1.18 (32).

### 3.2 Working memory deficits

Deficits in WM have been extensively reported in SCZ (6). Individuals with SCZ underperform controls on the Wechsler Memory Scale-III Letter-Number Sequencing task—which involves mentally reordering mixed numbers and letters. This deficit is independent of their clinical state. FDRs typically display an intermediate performance, scoring significantly higher than probands but lower than the healthy population (33). These data support the proposed role of WM deficits as a SCZ endophenotype (34). Furthermore, task-related functional studies have revealed dysfunctional fronto-striatal connectivity during the WM task in both individuals with SCZ (35) and unaffected siblings (36).

### 3.3 Verbal memory deficits

Deficits in verbal declarative memory (VDM) are well-documented in SCZ (6). These impairments appear independent of medication status and illness duration; however, evidence suggests an association with negative symptoms (37, 38). Consistent with other SCZ endophenotypes, VDM deficits have also been reported in FDRs and in subjects at high risk for psychosis (39). Furthermore, investigations targeting secondary verbal memory in both patients and their relatives have found that dysfunction in this domain correlates with negative symptoms—irrespective of illness duration or neuroleptic dosage—supporting its validity as a candidate endophenotype for SCZ (40).

## 4 Social cognition endophenotypes

SC represents an emerging field in SCZ research. This domain encompasses a set of dimensions that concern how social knowledge is structured and defined, as well as the processes underlying social decision-making and judgment (41). Currently, SC is conceived as the set of mental operations that subserve social interactions, enabling individuals to interpret and predict the behavior of others within dynamic social contexts (42). Given the growing prominence of this field, the Measurement and Treatment Research to Improve Cognition in Schizophrenia (MATRICS) initiative established a consensus to clarify the core concepts and criteria defining the construct (43, 44). This consensus identified four primary domains: Theory of mind (ToM), Emotion Processing (EP), Social Perception (SP), and Attribution Bias (AB) (42–44). Recent evidence indicates that SC deficits are not only characteristic of SCZ but are also observed in unaffected relatives and individuals at UHR for psychosis (45). Collectively, these findings suggest that SC may be an endophenotype of SCZ. The following sections review the evidence supporting these specific SC components as endophenotypes.

### 4.1 Theory of mind deficits

ToM refers to the ability to attribute mental states to others (45, 46). The link between SCZ and ToM has primarily been discussed in the context of Frith’s work (47), who proposed that SCZ involves a deficit in metarepresentational skills. This deficit leads to a failure in the mentalization process and an inability to think about one’s own and others’ mental states (42). In recent years, a growing body of evidence has suggested that ToM may serve as an endophenotype for SCZ. Several studies have confirmed that ToM is impaired in SCZ, regardless of the assessment method used. Furthermore, it is considered a trait marker, remaining stable throughout the course of the illness (48, 49). Moreover, research suggests that ToM may be a potential predictor of functional outcomes in individuals with SCZ (49, 50). Consistent with the criteria for validating endophenotypes, studies focusing on ToM have reported deficits in unaffected relatives compared to healthy individuals (51, 52). However, some meta-analyses have reported only modest effect sizes for ToM deficits in non-affected relatives (53, 54). Similarly, studies on UHR subjects have found modest ToM deficits compared to healthy controls (53). Conversely, another study observed lower ToM scores in UHR individuals who transitioned to psychosis compared to those who did not (55). Interestingly, recent studies suggest that ToM abnormalities may help distinguish between first-episode SCZ patients and individuals at clinical or familial risk for psychosis (56).

### 4.2 Emotion processing deficits

This SC component concerns abilities for adaptive perception and the use of emotions (42). In SCZ, deficits in this area are particularly evident in the interpretation of facial expressions (36, 39). Concerning emotional regulation, studies have revealed severe impairments (45, 57, 58). However, research involving relatives of individuals with SCZ has yielded inconsistent results; while some meta-analyses report moderate effect sizes in first-degree relatives, others failed to detect such impairments (51, 59). Similarly, findings in high-risk populations appear inconsistent. Although some investigations have reported significant impairment in emotional abilities in UHR subjects during facial recognition and voice intonation tasks, longitudinal studies on affect recognition suggest that these deficits may not serve as a reliable marker of psychosis vulnerability (56, 60, 61).

### 4.3 Social perception and attributional bias deficits

Social Perception concerns the understanding of rules and roles necessary for effective interaction within different social contexts (42). Attributional Bias, conversely, involves the cognitive processes used to ascribe causes to the behavior of others, situational factors, or oneself (42, 62). Research findings regarding Social Perception are less consistent than those for ToM. While individuals with SCZ reportedly exhibit deficits in identifying negative emotions such as anger, fear, and sadness, these subjects did not differ from healthy controls in their ability to infer complex personality traits (63). In contrast, a meta-analysis demonstrated a medium-to-large deficit in Social Perception among individuals with SCZ (64). Regarding Attributional Bias, investigations consistently indicate that patients with SCZ tend to attribute adverse events to external causes; this pattern is particularly pronounced in patients with paranoid delusions (45, 65). However, other studies suggest that individuals with SCZ may exhibit a tendency toward internal attributions for adverse events, displaying a self-blaming bias (66).

Regarding Social Perception in risk populations, evidence in relatives of individuals with SCZ is more limited and inconsistent. While some studies report moderate deficits in relatives, others indicate that parents of patients with SCZ may perform better than controls (51, 54, 67). Similarly, findings in high-risk subjects are mixed; meta-analyses have reported a small effect size for social perception impairments in UHR subjects (68, 69).

Concerning Attributional Bias, data in first-degree relatives remain inconclusive, although more recent studies reported that these individuals exhibited an altered Attributional Bias compared to controls (59). In high-risk subjects, findings are also mixed: some studies indicate that UHR individuals score higher on external bias than controls (70), whereas other reports observe no significant differences (71).

## 5 Discussion

SCZ has a heritability of 60-80%, much of which is attributable to common risk alleles. Recently, in a two-stage exhaustive association study involving thousands of individuals with SCZ and controls, researchers reported associations with common variants at 287 genomic loci, concentrated in genes expressed in excitatory and inhibitory neurons of the central nervous system rather than in other tissues and cell types (72). Similarly, in a search for the genetic basis of endophenotypes, the Consortium on the Genetics of Schizophrenia (COGS-1) used factor analysis to determine the factor structure and heritability of neuropsychological and neurophysiological endophenotypes in a study that included SCZ probands, their non-psychotic siblings, and community comparison subjects. Notably, neuropsychological measures showed a consistent amount of shared variance, whereas neurophysiological measures showed unique contributions as endophenotypes for SCZ (73). While identifying genes associated with SCZ may help isolate biological pathways, investigating endophenotypes unveils components of liability narrower than those of conventional clinical methods for diagnosing SCZ, potentially contributing to the search for gene susceptibility and disease-related biological pathways (74).

Recently, endophenotypes have become a component of the emerging paradigm of *stratified psychiatry.* Here, the definition of a psychiatric disorder is reconceptualized using clinical signatures and a multidimensional understanding of disease signs and symptoms, enabling patient stratification (75). Conceptually, clinical signatures aim to represent the patient’s disease state at a given time along quantified dimensions in a multidimensional space, including signs, symptoms, and qualitative and quantitative measures such as biomarker or endophenotype expression. In this framework, biomarkers serve to identify specific inter-individual characteristics that predict treatment response (76). Therefore, stratified psychiatry allows for an increase in response and remission rates by assigning a patient to the right treatment already approved using a biomarker (endophenotype). Genome-wide association studies (GWAS), a relatively recent and innovative approach, have made significant discoveries: complex disorders like SCZ are polygenic, with a large number of copy variants (77). Moreover, a polygenic risk score (PRS) has been associated with cognitive performance in patients with SCZ and in high-risk patients (78). Indeed, an increased PRS is linked to impaired cognition in SCZ, and a large study identified 21 independent SCZ risk loci that may influence cognition (79). Significantly, a prominent group of SCZ-associated genes is enriched in medium spiny neurons and hippocampal projection neurons (80). Given that cognitive dysfunction is an integral part of SCZ, using cognition as a stratification criterion for patient selection can improve the understanding of its genetic architecture (78). Complementary imaging studies have confirmed substantial reductions in white matter homogeneity across most brain areas, identifying white matter pathology and functional dysconnectivity as key components of SCZ pathophysiology contributing to cognitive dysfunction. Thus, the combined use of cognition-related endophenotypes and neuroimaging shows great potential for patient stratification (78). The role of functional neuroimaging in detecting endophenotypes has become increasingly important. Recent evidence, supported by functional neuroimaging, indicates that ventral striatum-hippocampus coupling during reward processing represents a stratification endophenotype for psychotic disorders. This coupling is transdiagnostically associated with measures of positive symptoms and memory performance, while also showing familial aggregation linked to genetic risk for SCZ (81). These results can contribute to the development of therapeutic interventions, for example, targeting ventral striatum-hippocampus connectivity through neurofeedback or by indirect therapeutic interventions that enhance cognitive control in SCZ (81). As noted, symptoms and cognition are core features of SCZ. Negative symptoms and cognition represent critical tools leading to the stratification of SCZ patients. A large study reported that stratifying participants by the severity of negative symptoms over time showed that patients with moderate-to-severe, sustained negative symptoms had lower scores in learning, memory, and global functioning. These findings have important consequences for the treatment needs, especially for the group with sustained negative symptoms. This group may need specific treatment targeting cognitive impairment, such as cognitive remediation (82).

A perspective closely related to the preceding text concerns the transdiagnostic shift from categorical diagnoses to neurobiologically meaningful dimensions (83). Searching for endophenotypes in this dimensional approach has reinforced the value of some neurocognitive biomarkers like intelligence quotient and working memory across a spectrum, including SCZ, Bipolar Disorder I (BDI), and mixed primary psychosis (83), suggesting they could be candidates for shared molecular mechanisms. Moreover, working memory is conceptualized as an endophenotype encompassing SCZ and affective disorders like major depressive disorder and bipolar disorder. Imaging studies during an n-back task showed that, across all patient groups, blunted activity was observed in the striatum, anterior insula, and frontal lobe, confirming that the same brain networks supporting working memory are impaired in SCZ, major depressive disorder, and bipolar disorder. These results suggest common functional abnormalities across SCZ, and mood disorders related to working memory. Similarly, an investigation into social dysfunction has revealed that this alteration is associated with decreased functional connectivity in the rostromedial frontal cortex of the Default Mode Network (DMN), both in SCZ and Alzheimer’s disease. These findings support DMN as a transdiagnostic endophenotype (84). This transdiagnostic dimension entails a redefinition of the concept of endophenotype. This does not imply that the endophenotype concept should be discarded; rather, we should accept the transdiagnostic nature of genetic and neural vulnerabilities, considering interaction among multiple endophenotypes and complex functional behavioral outcomes (85). Further research must incorporate this transdiagnostic dimension and use multiple endophenotypes as a stratification tool in contexts that require progressive standardization to assess psychiatric symptoms and side effects, thereby evaluating the predictive value of treatment modalities across studies and across different endophenotypes (biomarkers) (76).

## 6 Conclusion

As discussed above, substantial evidence has been reported regarding neuropsychological and neurophysiological endophenotypes in SCZ research across stratified and transdiagnostic dimensions. Similarly, recent investigations demonstrate greater impairments in SCZ on mentalization tasks (e.g., false belief, humor, intentionality) compared to other psychiatric conditions. This highlights the involvement of SC in the psychosis spectrum and opens new avenues for targeted therapeutic interventions (86).
